# The Medical and Financial Burden of Illegal Abortion

**DOI:** 10.7759/cureus.30514

**Published:** 2022-10-20

**Authors:** Grecia Rivera Rodriguez, Jean Tamayo Acosta, Ariel E Sosa Gomez, Rosymar E Marcucci Rodriguez, Gissete A Rodriguez Cintron, Marjorie Acosta

**Affiliations:** 1 General Internal Medicine, University of Puerto Rico, Medical Sciences Campus, San Juan, PRI; 2 Medicine, University of Medicine and Health Sciences, Bayamon, PRI; 3 Psychiatry, Saint James School of Medicine, Miami, USA; 4 Internal Medicine, University of Puerto Rico, Medical Sciences Campus, San Juan, PRI; 5 Psychiatry, Hospital Pavia Hato Rey, Bayamon, PRI

**Keywords:** health-care policy, health policy making, health policy and advocacy, financial impact, roe v. wade, illegal abortion

## Abstract

More than a heated debate subject, abortion is a matter that has been present in human history for a very long time. As our society evolves and advances in medicine and socioeconomic systems are made, the subject of the medical procedure known as abortion appears to be a differentiator in our behaviors as a society. This article highlights the known effects and medical complications of illegal abortion and the financial impact of the procedure's legal status. A retrospective search using EBSCO, PubMed/Medline, Cochrane, EMBASE: Excerpta Medica Database, and DARE electronic databases was conducted, focused on detailing the risks of illegal abortion, the financial cost of complications, the socioeconomic impact of unwanted progeny, and the rationale behind seeking the procedure, legally or otherwise. Each author independently reviewed and extracted data to write up each assigned section, and group collaborations occurred to create the final draft. Out of the 87 resources reviewed, 16 sources were deemed eligible for this article, and their data are herein outlined.

## Editorial

Introduction

When not managed by a medical professional, abortion is a risky and costly procedure. When abortion was illegal in the United States, individuals resorted to illegal means of attaining it. These included but were not limited to foreign bodies placed in the uterus through the cervix, such as sticks dipped in oil, wire, knitting needles, coat hangers, ballpoint pens, and air blown in by either a syringe or turkey baster. While illegal abortions carry inherent risks, as per the World Health Organization (WHO), the main causes of death from unsafe abortion are hemorrhage, infection, sepsis, genital trauma, and necrotic bowel [1]. While this article does not aim to cover the potential cost of all complications of abortion, we would like to illustrate the cause of treating just one of them, sepsis. In the 1970s, before Roe v. Wade set a legal precedent, complications of unsafe, illegal abortions were common occurrences in medical wards, and the healthcare system was taxed with treating these preventable situations [2]. As we transition to an era of uncertainty with the overturning of Roe v. Wade, it is important to learn from our history to avoid repeating prior mistakes.

Financial impact of abortion

Note that in the present day, the cost of treating a septic patient can go as high as $51,000 per case [3]. Factoring in that a total of 629,898 legal abortions were reported to CDC from 48 reporting areas in 2019 [4], even if a fifth of these women had attempted an illegal abortion for whichever reason and subsequently developed the due complications; it would have cost upwards of 6.4 billion dollars to attempt saving the lives of these women in 2019 alone. In addition to sepsis, hemorrhage, uterine perforation, and internal organ damage, all of which are medical emergencies, are likely complications of illegal abortion that carry a heavy management cost and often end in the patient’s death.

From a financial perspective, the cost of caring for uninsured patients falls on hospitals and taxpayers. Please note that before Roe vs. Wade, individuals of low socioeconomic status and those from minority groups who could not access abortion safely turned to various unsafe methods to meet their needs. Methods include, but are not limited to self-medication with toxic chemicals such as turpentine, bleach, detergent solutions, quinine, and strong teas [2], all of which are now treated at our emergency departments. The compounding economic costs of banning abortion are then something that must be taken into consideration, for they are significant.

Hospitals used to have wards dedicated exclusively to the treatment of illegal abortions; these wards, alongside the enormous number of medical and surgical complications and noted emotional traumas associated with illegal abortion and its complications, all disappeared after the installment of Roe vs. Wade [5,6]. The mean total cost per patient with abortion or post-abortion care ranged from 23 USD to 564 USD. The annual costs ranged from 189,000 USD to 134 million USD in 2019. With an increased rate of non-medically managed abortions, the cost of caring for patients with complications from said abortions is bound to make that phrase pale in comparison to the billions of dollars that will be needed to support the health of our citizens.

Legal versus illegal abortion

It is a fact that abortion, the documentation of which can be traced back to 2700 BC, is a fundamental part of human history; making it illegal is unlikely to make it go away [2]. Even in the modern day, the incidence of securing an abortion in countries that restrict abortion and those with more liberal access is interestingly equivalent [7-9]. As per the CDC [4], the incidence of abortion is 11.4 per 1000 women in the US alone. According to data from statistical studies, the legality of abortion across the world has little to no effect on abortion rates worldwide. Legal or not, abortions can, will, and do take place. The legality of abortion, however, does affect how safe those abortions are [10].

The question arises as to why individuals decide to undergo abortions and what happens when they are denied a legal means to obtain one. Research has shown that women who were denied an abortion and successfully gave birth were more likely than women who received an abortion to experience economic hardship and financial insecurity lasting years [11], thus raising the country’s poverty index.

While many women may abort because of their current circumstances, they often proceed to want to have children upon the improvement of said circumstances; the medical complications of an illegal abortion are likely to place the patient in a position where even if they wanted to have children, they would be physically unable to; or at the very least, the risk of pregnancy would be immeasurably fatally dangerous to both mother and fetus. Worldwide, an estimated 68,000 women (eight/hour) die from an illegal abortion. This translates to a case-fatality rate of 367/100,000, which is hundreds of times greater than legal abortions [2]. In essence, abortions will happen, whether they are legal or not. Illegal abortions, however, carry a much heavier toll, both medically and financially, as compared to legal ones. Table 1 summarizes the findings of the studies featured in this article.

**Table 1:** Summary of research articles detailing the impact of legal and illegal abortion.

Study	Authors	Article class	Summary
Life without Roe v Wade	Ginsberg and Shulman [2]	Commentary	To reacquaint women and physicians as to what to expect if or when abortion becomes illegal.
Epidemiology and costs of sepsis in the United States: an analysis based on timing of diagnosis and severity level	Paoli et al. [3]	Retrospective observational study	Costs of treating sepsis ranged from $39,336 in sepsis without organ dysfunction to $68,671 for septic shock per case.
Abortion surveillance - United States, 2019 [4]	Kortsmit et al. [4]	Meta-analysis	CDC conducts abortion surveillance to document the number and characteristics of women obtaining legal induced abortions and number of abortion-related deaths in the United States. Among the 49 reporting areas that provided data for 2019, a total of 629,898 abortions were reported.
Repercussions of overturning Roe vs. Wade for women across systems and beyond borders	Coen-Sanchez et al. [5]	Editorial	Authors discuss the impact of overturning Roe vs Wade and how the decision affects the reproductive health of women.
Abortion before and after Roe	Tan et al. [6]	Review article	Abortion policies could have a significant impact on the availability of services in other states in which physicians performing abortions lack admitting privileges at nearby hospitals.
Unsafe abortion: the preventable pandemic	Grimes et al. [7]	Series	The availability of modern contraception can reduce but never eliminate the need for abortion.
New estimates and trends regarding unsafe abortion mortality	Ahman and Shah [8]	Review article	1 in 8 maternal deaths globally and 1 in 5 maternal deaths in Eastern Africa continue to be attributable to unsafe abortion.
Unsafe abortion in 2008: global and regional levels and trends	Shah and Ahman [9]	Review article	47,000 women per year are estimated to lose their lives from the complications of unsafe abortion.
Unsafe abortion: global and regional incidence, trends, consequences and challenges	Shah and Ahman [10]	Review article	Legal restrictions on abortion force women into seeking abortion from non-trained individuals or under non-hygienic condition which causes and increase in complications and even death.
Socioeconomic Outcomes of women who receive and women who are denied wanted abortions in the United States	Foster et al. [11]	Review article	Legalization of abortion creates a decline in childbearing, which improves women educational and economical progress.
The macroeconomics of abortion: a scoping review and analysis of the costs and outcomes	Rodgers et al. [12]	Review article	At the macro level, post-abortion care services are expensive and can constitute a substantial portion of government health budgets. Public sector coverage of abortion costs is sparse, and abortion seekers bear most of the financial costs.
Characteristics of US abortion patients, 2008. New York: Guttmacher Institute	Jones et al. [13]	Survey report	The profile of the women who gets an abortions is predominantly found to have a low-income, in his 20’s and unmarried.
Characteristics of women obtaining induced abortions in selected low-and middle-income countries	Chae et al. [14]	Review article	Wealthier women are more likely to report an abortion in 12 countries (three in Africa, seven in Asia, one in Europe, and one in Central America).
The long-term impact of restricted access to abortion on children's socioeconomic outcomes	Hajdu and Hajdu [15]	Retrospective observational study	During the restrictive Hungarian abortion policy in 1974, the law had a negative impact on the socioeconomical outcomes in children of mothers who were denied an abortion, which resulted in an increased chance of unemployment and probability of been a teen parent.
Comparison of health, development, maternal bonding, and poverty among children born after denial of abortion vs after pregnancies subsequent to an abortion	Foster et al. [16]	Longitudinal observational study	Abortion access gives women the choice of when having children, which results in more financial and emotional resources.
Contraception and abortion knowledge, attitudes and practices among adolescents from low and middle-income countries: a systematic review	Munakampe et al. [17]	Systematic review	Adolescents with higher education were more likely to opt for abortion in order to secure a proper education, and higher economical and social standing.

Sequelae of banning legal abortion

A set of scoping reviews from 2021 indicates that abortion regulations affect women’s education, participation in the labor market, and positive contribution to the growth of their countries' domestic product (GDP) improvement. Furthermore, the legal status of abortion can also affect children’s educational outcomes and their earnings in the labor market later in life [12]. The overall socioeconomic impact of abortion warrants analysis because when determining the most common reasons why women seek abortions, research has found that, throughout various countries, socioeconomic concerns were the most frequently cited cause of seeking abortion [13]. While we found that some articles [14] argue that women in better socioeconomic standing are more likely to seek legal abortions, it is of note that if the poverty index of a country rises, as it has been described herein, the incidence of individuals in a low socioeconomic position is bound to rise as well. Thus, raising the rates of illegal abortions and their subsequent medical complications precipitates a vicious cycle of financial and medical burden on the country. A research study examined the consequences of restricting abortion following a change in policy in Hungary in 1974. In 1974, restricted abortion rules were introduced to these populations to increase fertility. Access to abortion was restricted to the following groups: unmarried women, women with three or more children, and women living in poverty. A fee for women seeking an abortion for nonmedical reasons was charged. Between 1973 and 1974, the number of abortions decreased (169,650 to 102,022), and the number of live births increased by 30,000. However, evidence suggests that the number of illegal abortions could affect the number of live births [15].

A recent study suggested that abortion restrictions decreased educational achievement in children born after the law was implemented. These children were less likely to have a higher education degree and often classified themselves as unemployed [16]. The study also observed an increased probability of these children having offspring of their own before the age of 18 and a decreased probability of owning a property. Essentially, being an undesired child is directly proportional to having a low education level, which will eventually affect the socioeconomic status of the children affected by the abortion law.

A prospective study performed in the US studied the association between abortion access and children's outcomes. In this study, children born from pregnancies where women denied abortion were compared to subsequent children born after abortion in terms of future socioeconomic outcomes. This study found that children of mothers who were denied abortion were more likely to report insufficient resources to cover basic needs when compared to children born after an abortion [16]. Furthermore, children of mothers who were denied abortion were more likely to exhibit less bonding and a higher probability of injuries (possibly due to abuse or neglect). Although these findings were less significant, the study showed that women who had the choice to abort could better assess when they were financially and emotionally stable enough to fully care for their child. A systematic review of various studies found that adolescents who underwent abortion felt that their future was protected by securing education and were granted economic and social empowerment, which they attributed to having had the opportunity to decide when to have a child [17]. It was observed that adolescents with better educations had more abortions as compared to those with less privileged educations. The potential impact of socioeconomic status, however, may not be taken out of consideration.

Conclusion

Abortion, legal or not, is an intrinsic part of human behavior and, as proven by our history, individuals are likely to seek even unsafe means to obtain the desired result. While we might not strive to control human behaviors, it is possible to rationally measure the effects illegal abortion has on society. Access to safe medical procedures is a human right regardless of personal morals or opinions. This article explored how the legality of abortion impacted both the United States and other countries with different cultures and laws. While it is not our aim as authors to incite unnecessary debate in society, we have found that we, as a community, must fully and objectively evaluate the impact that overturning a human right will have on us in the future. Further research is encouraged regarding how to best respond to the complications of illegal abortion in order to minimize the toll it will take on society.
